# Supplementary material for: LPS-Dephosphorylating Cobetia amphilecti Alkaline Phosphatase of PhoA Family Divergent from the Multiple Homologues of Cobetia spp
Source: Microorganisms. 2024 Mar 21;12(3):631. doi: 10.3390/microorganisms12030631 (PMC10974088; doi:10.3390/microorganisms12030631)
Supplement: Supplementary file 1 [file microorganisms-12-00631-s001.zip › Figure S1 Electrophoresis.pdf]

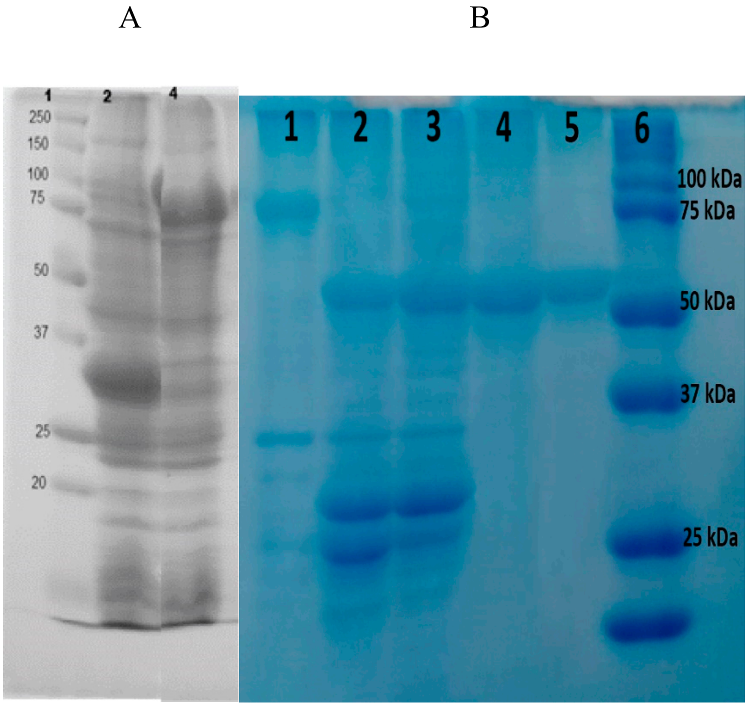

Figure S1. **A** - Electrophoregram of protein lysates from the recombinant cultures *E. coli* Rosetta (DE3)/Pho40: lanes1 – the protein weight markers for electrophoresis, kDa (BioRad, USA); lane 2 – the cell extract from the control strain *E. coli* Rosetta (DE3)/pET-40b(+) after IPTG-induced expression (a band corresponding to a molecular mass ≈ 30 kDa is of the empty plasmid chaperon DsbC); lane 4 – the cell extract from the recombinant colonies of *E. coli* Rosetta (DE3)/Pho40 after IPTG-induced expression (the band corresponding to a molecular weight ≈ 90 kDa are the recombinant hybrid proteins DsbC/CmAP). **B** - Electrophoregram of the recombinant alkaline phosphatase CmAP at the different stages of purification: lane 1 - after metal affinity chromatography (≈ 90 kDa); lanes 2-3 – after removing the plasmid chaperon due to incubation with enteropeptidase L-HEP (≈ 55 kDa); lanes 4-5 - after ion exchange chromatography (≈ 55 kDa); M- the protein weight markers for electrophoresis, kDa (BioRad, USA).
