# Supplementary material for: LPS-Dephosphorylating Cobetia amphilecti Alkaline Phosphatase of PhoA Family Divergent from the Multiple Homologues of Cobetia spp
Source: Microorganisms. 2024 Mar 21;12(3):631. doi: 10.3390/microorganisms12030631 (PMC10974088; doi:10.3390/microorganisms12030631)
Supplement: Supplementary file 1 [file microorganisms-12-00631-s001.zip › Table S2 ALP content.pdf]

Table S2. The content and distribution of alkaline phosphatase families in *Cobetia* spp. isolates

Original Strain title	Actual species	Accession ID	ID protein	ALP family	Isolation source
C. amphilecti KMM 296*	*C. amphilecti*	GCF_000754225.1	WP_043332298.1	PafA	Coelomic fluid of mollusc *C. grayanus* (Russia: The Sea of Japan)
			WP_043333989.1	PhoD	
			WP_245163010.1	**PhoA***	
			WP_043336117.1	PhoX	
Cobetia sp. UCD-24C*	*C. amphilecti*	GCF_001306765.1	WP_052384691.1	PhoD	Seagrass *Zostera* sp. sediment
			WP_082388216.1	PhoD	
			WP_054555671.1	PhoD	
			WP_054555885.1	PafA	
			WP_235507008.1	**PhoA***	
			WP_054557251.1	PhoX	
C. amphilecti B2M13*	*C. amphilecti*	GCF_018860945.1	WP_244994940.1	PhoD	Alginate 40-100 m particle (artificial) (USA: Canoe Beach)
			WP_234368129.1	**PhoA***	
			WP_216060785.1	PhoX	
			WP_216060931.1	PafA	
			WP_216061230.1	PhoD	
Cobetia sp. 1AS1*	*C. amphilecti*	GCF_029846435.1	WP_279838219.1	PafA	Coastal seawater (Russia: the Sea of Japan, Vostok Bay)
			WP_279838543.1	**PhoA***	
			WP_279838774.1	PhoX	
			WP_279839461.1	PhoD	
			WP_279839645.1	PhoD	
Cobetia sp. 1CM21F*	*C. amphilecti*	GCF_023161745.1	WP_248377282.1	**PhoA***	Sea cave (Portugal: Algarve)
			WP_248377879.1	PhoX	
			WP_248378997.1	PafA	
			WP_248380112.1	PhoD	
			WP_248380900.1	PhoD	
C. amphilecti NRIC 0815^{T}*	*C. amphilecti*	GCA_030010415.1	WP_284726718.1	PhoD	The finger sponge *A. digitatus* (The Sea of Okhotsk, Sakhalin Island, Piltun Bay)
			WP_284726808.1	**PhoA***	
			WP_284726995.1	PhoX	
			WP_284727213.1	PhoD	
			WP_284727576.1	PafA	
Cobetia sp. AM6*	*C. amphilecti*	GCF_009617955.1	WP_172978613.1	PhoD	Exterior surface of the shell of an abalone sold in a fish market (Tokyo, Japan)
			WP_153635520.1	PafA	
			WP_153635957.1	PhoX	
			WP_242007665.1	**PhoA***	
			WP_153636482.1	PhoD	
Cobetia sp. 2AS1	*C. amphilecti*	GCF_014876835.1	WP_192837404.1	PhoD	Coastal sediment (Russia: the Sea of Japan, Nakhodka harbor)
			WP_192838056.1	PafA	
			WP_225996507.1	PhoD	
			WP_192839216.1	PhoX	
Cobetia sp. 2AS	*C. amphilecti*	GCF_029846355.1	WP_192838056.1	PafA	Sediments (Russia: the Sea of Japan, Vostok Bay)
			WP_192839216.1	PhoX	
			WP_225996507.1	PhoD	
			WP_192837404.1	PhoD	
C. litoralis NRIC 0814^{T}	*C. amphilecti*	GCF_029846315.1	WP_249330383.1	PhoD	Sandy sediment (Russia: the Sea of Japan)
			WP_279830791.1	PafA	
			WP_279833222.1	PhoX	

Cobetia sp. MC34	*C. amphilecti*	GCF_018340035.1	WP_279832006.1<br>WP_249330383.1<br>WP_213114436.1<br>WP_213114659.1	PhoD<br>PhoD<br>PhoX<br>PhoD	Fish-landing facility (Norway: Troms, Tromvik)
Cobetia sp. 29-18-1	*C. amphilecti*	GCF_029846405.1	WP_279835410.1<br>WP_279835708.1<br>WP_279836670.1<br>WP_279836807.1	PhoD<br>PhoX<br>PafA<br>PhoD	The finger sponge *Amphilectus digitatus* (The Sea of Okhotsk, Sakhalin Island, Piltun Bay)
Cobetia sp. 4B	*C. amphilecti*	GCF_018831605.1	WP_215824272.1<br>WP_240548688.1<br>WP_215822940.1<br>WP_215823407.1	PafA<br>PhoD<br>PhoX<br>PhoD	Current Humbolt system, *Heterostera chilensis* (Chile: Puerto Aldea)
C. amphilecti N-80	*C. amphilecti*	GCF_020217465.1	WP_225347537.1<br>WP_225347908.1<br>WP_225346724.1<br>WP_225347230.1	PhoD<br>PafA<br>PhoX<br>PhoD	The marine sediment (Atlantic Ocean: Scotia Sea)
Cobetia sp. Dlab-2-U	*Cobetia* sp.	GCF_024124585.1	WP_253061861.1<br>WP_253063154.1<br>WP_253064500.1<br>WP_253064933.1	PafA<br>PhoD<br>PhoX<br>PhoD	Coral surface mucus layer and tissue, *Diploria labyrinthiformis* (USA: FL, Dry Tortugas)
Cobetia sp. Dlab-2-AX	*Cobetia* sp.	GCF_024124625.1	WP_253061861.1<br>WP_253063154.1<br>WP_253064500.1<br>WP_253064933.1	PafA<br>PhoD<br>PhoX<br>PhoD	Coral surface mucus layer and tissue, Diploria labyrinthiformis (USA: FL, Dry Tortugas)
C. marina 402	*Cobetia* sp.	GCF_013350055.1	WP_175089240.1<br>WP_175090103.1<br>WP_254627190.1<br>WP_175090544.1	PhoD<br>PhoX<br>PhoD<br>PafA	Aquarium water (Brazil: Rio de Janeiro)
Cobetia sp. cqz5-12	*Cobetia* sp.	GCF_016495405.1	WP_233593417.1<br>WP_200019524.1<br>WP_200016943.1<br>WP_200017549.1	PhoD<br>PafA<br>PhoX<br>PhoD	Rotten brown algae, Sargassum fusiforme (China:Zhejiang)
Cobetia sp. MB87	*Cobetia* sp.	GCF_011319755.1	WP_240916767.1<br>WP_166020003.1	PhoD<br>PhoD	Sea cucumber gut (Viet Nam: Hon Tre, Nha Trang Bay)
C. pacifica GPM2	*C. marina*	GCF_009931455.1	WP_084208519.1<br>WP_254696557.1<br>WP_254696414.1	PhoX<br>PhoD<br>PhoD	Namhae, the red algae Pyropia tenera (South Korea)
Cobetia sp. 10Alg 146	*C. marina*	GCF_029846385.1	WP_279854649.1<br>WP_279856192.1<br>WP_279856235.1	PhoX<br>PhoD<br>PhoD	The red algae Ahnfeltia tobuchiensis (Russia: The Sea of Okhotsk, Kuril Isles)
Cobetia sp. 3AK	*C. marina*	GCF_029846335.1	WP_240499655.1<br>WP_279874635.1<br>WP_084208519.1	PhoD<br>PhoD<br>PhoX	Coastal seawater (Russia: The Sea of Japan, Vostok Bay)
C. pacifica NRIC 0813^{T}	*C. marina*	GCA_030010515.1	WP_284728477.1<br>WP_240704267.1<br>WP_084208519.1	PhoD<br>PhoX<br>PhoX	Sandy sediment (Russia: the Sea of Japan)
Cobetia sp. MMG027	*C. marina*	GCF_027947415.1	WP_271302424.1<br>WP_084208519.1	PhoD<br>PhoX	missing (USA: Mission Beach, San Diego)

C. marina MM1IDA2H-1AD	*C. marina*	GCF_900119965.1	WP_240499655.1	PhoD	Eulitoral intertidal pond at sea level (Chile: Valparaiso)
			WP_077375791.1	PhoX	
			WP_255303155.1	PhoD	
			WP_258038356.1	PhoD	
Cobetia sp. 5-11-6-3	*C. marina*	GCF_013374055.1	WP_255303113.1	PhoD	Seaweed (Japan: Iwate, Ofunato Bay)
			WP_255303155.1	PhoD	
			WP_084208519.1	PhoX	
C. marina T1	*C. marina*	GCF_005144735.1	WP_240704252.1	PhoD	Salt water (USA: New Hampshire, Hampton Beach)
			WP_240704267.1	PhoD	
			WP_136939347.1	PhoX	
C. marina NBRC 15607	*C. marina*	GCF_006540105.1	WP_136939347.1	PhoX	missing
			WP_240704267.1	PhoX	
Cobetia sp. 5-25-4-2	*C. marina*	GCF_013374075.1	WP_255304446.1	PhoD	Seaweed (Japan: Iwate, Ofunato Bay)
			WP_255304452.1	PhoD	
			WP_176494061.1	PhoX	
Cobetia sp. ICG0124	*C. marina*	GCF_004006355.1	WP_127734507.1	PhoD	The green algae Ulva (United Kingdom: Aberystwyth)
			WP_084208519.1	PhoX	
			WP_240665388.1	PhoD	
C. marina JCM 21022^{T}	*C. marina*	GCF_001720485.1	WP_240499655.1	PhoD	Littoral water sample (USA: Woods Hole, MA)
			WP_084208519.1	PhoX	
			WP_240499593.1	PhoD	
Cobetia sp. L2A1	*Cobetia* sp.	GCF_009796845.1	WP_233266401.1	PhoD	Beach, brown algae (Arctic Ocean)
			WP_024952594.1	PhoX	
			WP_158773983.1	PafA	
			WP_158774142.1	PhoX	
			WP_158774696.1	PhoD	
Cobetia sp. QF-1*	*C. crustatorum*	GCF_002213105.1	WP_246116512.1	**PhoA***	Seawater (China: Qingdao)
			WP_088743230.1	PhoD	
			WP_088743763.1	PhoX	
			WP_088743908.1	PhoX	
C. crustatorum SM1923*	*C. crustatorum*	GCF_007786215.1	WP_144726746.1	PhoD	Surface seawater (Kongsfjorden, Arctic)
			WP_088743763.1	PhoD	
			WP_144727163.1	PhoX	
			WP_246116512.1	**PhoA***	
			WP_144728015.1	PhoD	
C. crustatorum JO1^{T}*	*C. crustatorum*	GCF_000591415.1	WP_248623642.1	**PhoA***	Fermented shrimp (South Korea: Daejeon)
			WP_282705494.1	PhoD	
			WP_282705495.1	PhoD	
			WP_024952594.1	PhoX	

* The strains containing **PhoA** family.
