# Supplementary figures and images for: LPS-Dephosphorylating Cobetia amphilecti Alkaline Phosphatase of PhoA Family Divergent from the Multiple Homologues of Cobetia spp
Source: Microorganisms. 2024 Mar 21;12(3):631. doi: 10.3390/microorganisms12030631 (PMC10974088; doi:10.3390/microorganisms12030631)

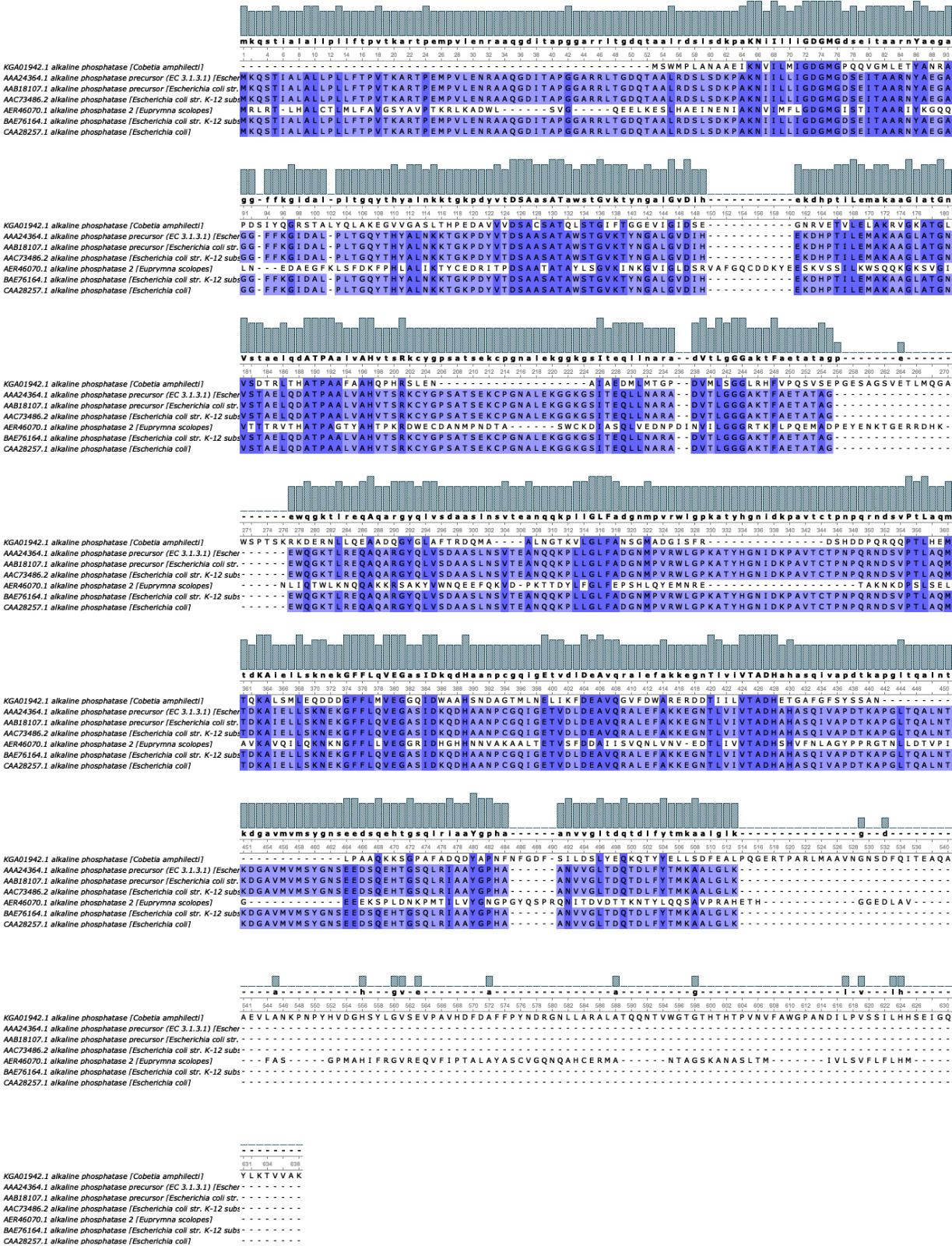

Supplement: Supplementary file 1 [file microorganisms-12-00631-s001.zip › Figure S2 Multiple alignment.jpg]
